# Cutaneous histiocytic sarcoma arising from soft tissue filler injections: a case report

**DOI:** 10.3389/fonc.2025.1403881

**Published:** 2025-03-28

**Authors:** Jangyoun Choi, Heeyang Park, Eun Jeong Ko, Jong Yun Choi, Suk-Ho Moon, Deuk Young Oh, Young-Joon Jun

**Affiliations:** Department of Plastic and Reconstructive Surgery, Seoul St. Mary’s Hospital, The Catholic University of Korea, Seoul, Republic of Korea

**Keywords:** histiocytic sarcoma, foreign-body reaction, fillers, tumorigenesis, injection site reaction

## Abstract

**Background:**

The administration of soft-tissue fillers is a popular aesthetic procedure. Nonetheless, it can result in complications such as foreign-body reactions, infections, skin necrosis, granulomas, and even malignant transformation. This case study documents an unusual occurrence of sarcoma following a prior cosmetic injection.

**Case report:**

A male patient, aged 76, presented with facial swelling. He received filler injections from a non-professional aesthetician twenty-five years ago. The patient reported a slow downward movement of the injected material, recurring inflammation, and the development of multiple nodules. An excisional biopsy and tissue culture were conducted, which did not identify any microorganisms but did reveal a dense infiltration of large polygonal cells with pleomorphic nuclei. The diagnosis of histiocytic sarcoma(HS) was confirmed through immunohistochemistry. A comprehensive systemic examination, including contrast MRI and PET-CT, identified multiple nodular soft tissue lesions in the subcutaneous layer of the face and intense metabolic activity in the same nodular lesions. Metabolic activities were also observed in the abdominal wall, indicating a potential migration of the injected material. Following diagnosis, all remaining lesions in the forehead, nose, and abdomen were surgically excised. Due to the complete nature of the excision, adjuvant chemoradiation was not administered.

**Conclusion:**

This case underscores the serious complication that can result from unauthorized filler injections, including the extremely rare histiocytic sarcoma. It emphasizes the necessity of cautious follow-up and patient education in aesthetic procedures.

## Introduction

Soft-tissue filler injection is an essential part of cosmetic practice. Because of its high demand and case volume, its post-procedure follow-up is mainly neglected. However, serious complications can arise from a foreign-body reaction inciting local inflammatory reaction. The most common presentation is Cellulitis, secondary bacterial infection, skin necrosis, and granuloma. A chronic state of inflammation may trigger the malignant transformation of the injected soft tissue but is underreported. In this case report, we report an exceedingly rare case of primary cutaneous histiocytic sarcoma(HS) arising from a previous cosmetic injection. Distant filler migration was also observed, with concurrent development of the same disease.

## Case presentation

A 76-year-old male patient with diabetes first visited our clinic ten years ago with facial swelling. He had a history of filler injections on his forehead and both cheeks by an unlicensed individual twenty-five years ago. He had not been informed of the chemical properties of the filler. A few years after receiving the injection, the patient started to experience a gradual, descent of the injected material from the original area of treatment. Intermittent rubor and swelling of the injection site occurred repeatedly, which was only temporarily resolved with anti-inflammatory agents and oral antibiotics. The severity of symptoms continued to worsen, and the frequency of symptom occurrence increased. The patient visited our institution seeking surgical treatment for his condition.

On physical exam, multiple nodules were palpable along the bilateral nasolabial fold and forehead, which corresponded to the location of the past injections. The nodules were about 2-centimeter-sized, firm, and immobile. Since the lesion on the nasolabial fold area was the most disturbing, excision of the cheek nodules was planned. Under the impression of chronic foreign body reaction, excisional biopsy and tissue culture were performed.

**Figure 1:**
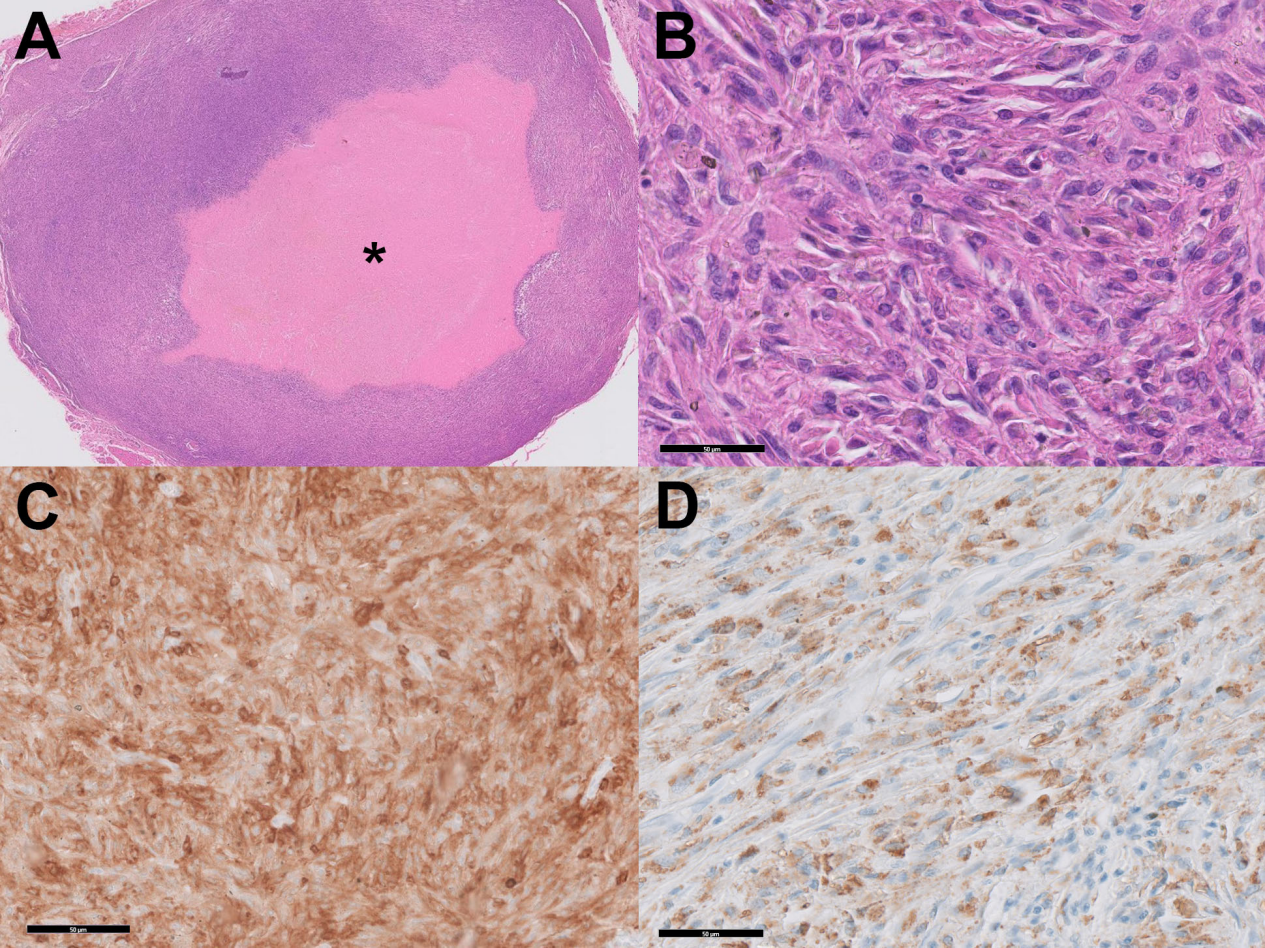
Histologic analysis. **(A)** Proliferation of histiocytes around the injected material shows a gel-pool-like accumulation (asterisk). **(B)** High-power magnification shows the epitheloid configuration, eosinophilic cytoplasm, and prominent nucleoli. (HE Stain, x400) **(C)** Immunohistochemical stain for CD4 shows diffuse immunoreactivity. **(D)** Immunohistochemical stain for CD68 shows positivity.

The tissue culture did not reveal any microorganisms. However, histology showed dense infiltration of large polygonal cells with epithelioid-to-pleomorphic morphology with pleomorphic nuclei, which needs to be differentiated from cancerous entities such as sarcoma (**Figures 1A, B**). Immunohistochemistry revealed high nuclear atypia, positivity in CD68 and CD15, and negativity in S100 and CD1a (**Figures 1C, D**). Based on these results, the diagnosis of HS was made.

**Figure 2:**
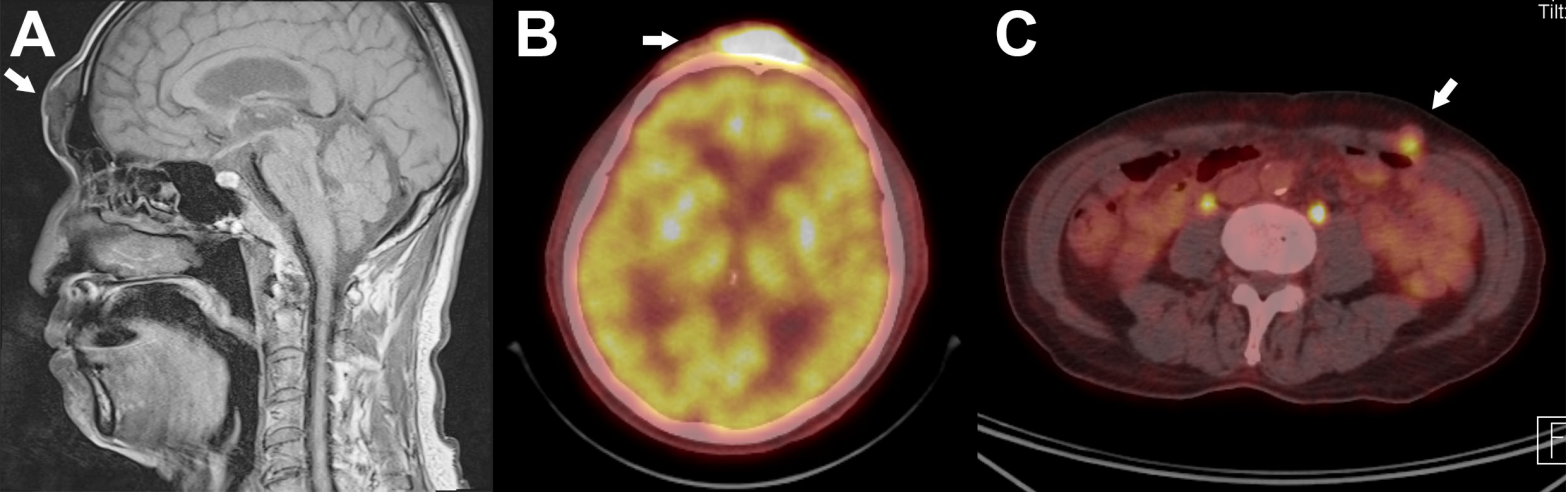
Magnetic Resonance Imaging(MRI) and positron Emission tomography(PET) Scan. **(A)** T1-Weighted sagittal view shows nodular accumulation of foreign body material in the subcutaneous layer of the forehead. **(B)** PET-CT scan shows high metabolic activity (SUVmax=15) in the same region as shown in **(A)**. **(C)** Hypermetabolic activity was also noted in the anterior abdominal wall, suggesting distant migration of the injected material. Biopsy confirmed the same pathology with the lesion in **(A)**.

Further systemic workup was performed. Contrast MRI showed multiple nodular soft tissue lesions in the subcutaneous layer of the glabella, nasolabial fold, and lower face. (**Figure 2A**) Positron emission tomogram computed tomography (PET-CT) showed intense metabolic activity in the same nodular lesions. (**Figure 2B**) Interestingly, metabolic activities were also noted in the abdominal wall, raising the possibility of migration of the injected material (**Figure 2C**).

**Figure 3:**
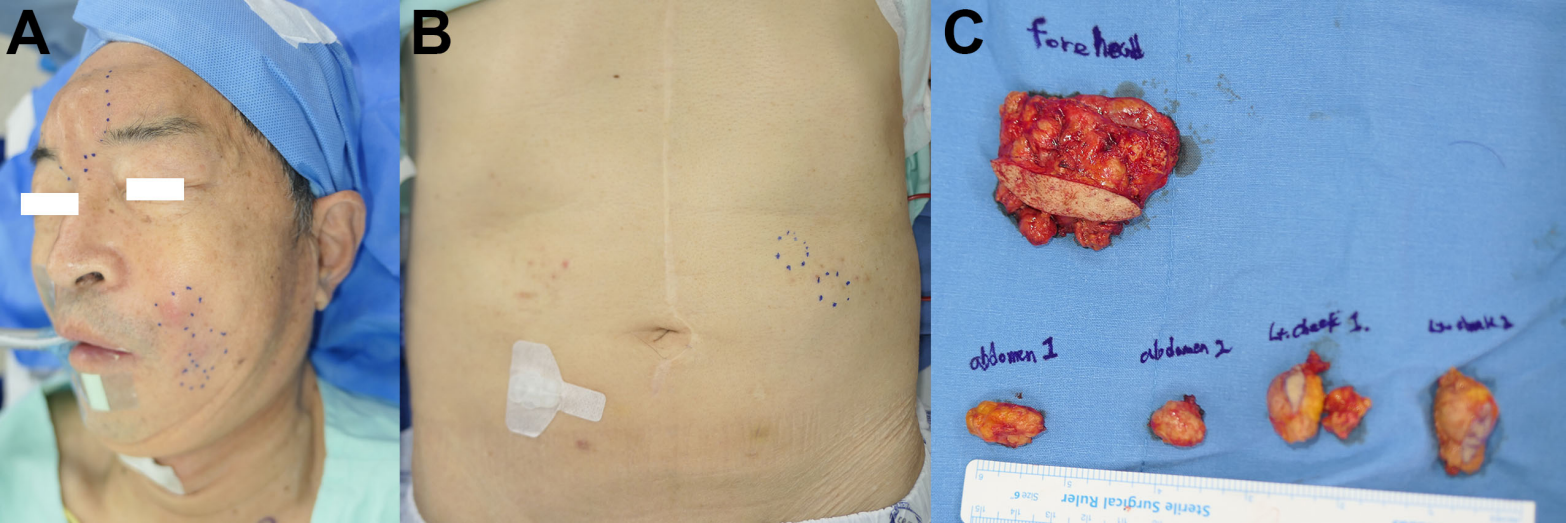
Clinical photo of the patient. **(A)** Enlarged nodular lesions in the nasolabial, forehead area are noted. The uppermost nodule in the nasolabial fold area shows erythematous change, indicating infection. **(B)** Similar nodular lesion in the abdominal wall suggests distant migration of the injected material. **(C)** Specimens resected *en bloc*.

**Figure 4:**
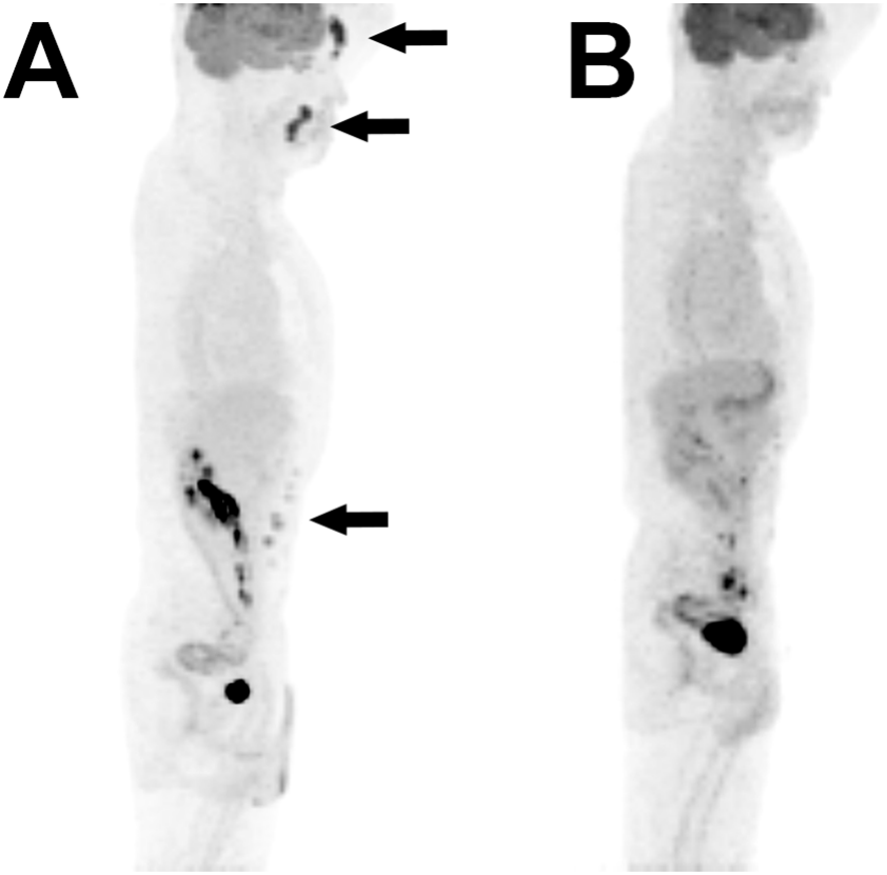
Pre- and post-operative PET scans. **(A)** Preoperative maximum intensity projection reconstituted PET scan shows increased metabolic activity in the forehead, nasolabial fold, and abdominal wall. **(B)** Postoperative PET image of the same reconstitution shows no activity in the corresponding regions.

After the confirmed diagnosis of histiocytic sarcoma, subsequent removal of all remaining lesions in the forehead, nasolabial area, and abdomen were performed (**Figures 3A-C**). Adjuvant chemoradiation was not administered owing to the complete nature of the excision. The patient is being followed up disease free, with thorough physical exams for unusual formation of nodules, and PET-CT scans at 6-month intervals (**Figure 4**).

## Discussions and conclusions

HS is an extremely rare non-Langerhans histiocyte disorder that presents as a unifocal or multifocal disease affecting various organ systems. Here, we presented an even more unique case of histiocytic sarcoma, which arose from a previously injected foreign material, which we think is the first to be reported.

Regardless of the location or chemical composition, malignancy arising from injected foreign material is possible but uncommon. The most relevant clinical scenario can be derived from the cosmetic injection of polyacrylamide hydrogel (PAAG), more commonly known as ‘AmazingGel’ or ‘Aquamid’, to the public (1). PAAG is a non-resorbable, synthetic material composed of approximately 2.5% cross-linked polyacrylamide and 97.5% water. It was widely used as an injectable filler for soft tissue augmentation, particularly for breast enhancement, from 1997 until its ban in 2006 (1). It has gained popularity due to its suggested biocompatibility due to its chemically hydrophilic nature. Through PAAG injection to the face and breast had been widely performed for augmentation purposes, Various complications followed, most related to inflammation, infection, asymmetry, and migration to other anatomical locations (2–4).

Few literature reported occurrence of malignancy after PAAG injection to the breast (5–7). Once considered biocompatible, questions were raised from its potential degradation into acrylamide, a probable human carcinogen. The exact carcinogenic mechanism of PAAG is not clearly identified, but *c-myc* overexpression may play a role (5, 8). When included in dietary form, exposure to acrylamide is well known to cause neurotoxicity, genotoxity, and carcinogenicity, and thus has been classified as group 2A substance by the International Agency for Research on Cancer (IARC) (9).

The injected material can migrate along the fascial tissue plane of the body, which is a hallmark of long-term complication in PAAG injections (3). Similarly, remote migration of the injected material to the abdominal wall was also found in our case. The reason for such a high migration rate of PAAG is thought to be originating from the initial high inflammatory response with the surrounding tissues, rather than formation of a capsule. This may decompose the normal tissue structures and subsequently make way for migration along tissue planes (10).

Due to its infrequent occurrence, the type of malignancy in our case is a noteworthy feature. Given the history of cosmetic injection, differential diagnosis usually points to benign conditions such granuloma or abscess. However in this case, high uptake values in PET CT suggested otherwise and led us to perform a thorough pathologic investigation. Pathogenesis of HS is unknown and has not been reported to be produced by a foreign material. Primary cutaneous HS is exceedingly rare and occupies a minor proportion of all HS etiologies. In theory, longstanding deposition and cellular aberration of histiocytes around the foreign material may have played a role in tumorigenesis of the neighboring tissue, as has also been suggested in animal models (11). Rather than systemic progression, a multifocal but circumscribed development of HS in spots where the injection was performed supports this claim.

HS is notable for its broad overlapping features with other myeloid neoplasms (12). Its major differential diagnoses include anaplastic large-cell lymphoma, myeloid sarcoma, and Langerhans cell histiocytosis (13). Immunohistochemistry is vital in differential diagnosis, especially positive expression in CD68, CD163, lysozyme, CD4, and CD45. Expression of at least 2 of the above markers has been recommended in diagnosing HS from other differentials (14). Similarly, our case showed positive expression in CD68 and CD4, which fulfills the diagnostic criteria. In HS, negative expression for CD1a (for Langerhans cell) and CD35(for follicular dendritic cell) is expected, which also parallels the immunohistochemical marker expression of our case.

Due to its rarity and aggressive clinical course, there is currently no standard treatment regimen for patients with HS (15). Extracutaneous HS especially presents at an advanced stage and shows limited response to chemoradiation (13). However, some reports claim that primary cutaneous HS may show a better prognosis than other types of extranodal HS if complete resectability is guaranteed (16, 17). Similarly, we achieved complete resection in all lesions localized in PET-CT, and the patient is showing excellent disease course without adjuvant therapy. To establish a more confident treatment plan for this rare disease, a multidisciplinary approach including pathology, hematology, and radiation oncology is crucial (18). The decision not to start adjuvant therapy was made by the interdisciplinary tumor board team, which was guided by the achieved complete resection, absence of progressive lesions, and the long-standing localized nature of the sarcoma. Until now, the patient is remaining disease-free with meticulous physical examination and imaging.

## Conclusion

This case is the first reported instance of histiocytic sarcoma induced by foreign material, emphasizing the need for careful observation of the disease’s progression through long-term follow-up. The association between soft tissue augmentation procedures and the development of sarcomas is not well-established, making this case particularly noteworthy.

## Data Availability

The original contributions presented in the study are included in the article/supplementary material. Further inquiries can be directed to the corresponding author.
